# An Overview of Sex-Based Differences in the Onset and Progression of DKD in the Well-Known Model, ZSF1 Rats

**DOI:** 10.3390/life15101627

**Published:** 2025-10-18

**Authors:** Arunita Chatterjee, Sharma S. Prabhakar

**Affiliations:** Department of Internal Medicine, Texas Tech University Health Sciences Centre, Lubbock, TX 79430, USA; arunita.chatterjee@ttuhsc.edu

**Keywords:** DKD, proteinuria, hyperglycemia, glucose, lipid, rat model, disease progression, blood pressure

## Abstract

A better understanding of diabetic kidney disease (DKD) will help optimize its management. Few animal models replicate human DKD characteristics as closely as ZSF1 male rats. To address the male-specific focus in murine model systems, we aimed to characterize the manifestation of DKD in ZSF1 females and compare them with ZSF1 males and control rats (CD). ZSF1 males become obese at an early age. ZSF1 females are fatter and heavier than CD females but remain smaller, lighter, and more active than ZSF1 males throughout their lives. Male, but not female, ZSF1 rats become hypertensive with age. ZSF1 females have a higher heart rate in early life, which reduces significantly with age. ZSF1 males exhibit significant hyperglycemia from an early age. In contrast, female ZSF1 are not overly hyperglycemic; however, their blood glucose levels trend higher than those of CD females, and the difference is statistically significant. Both ZSF1 males and females develop progressive proteinuria. ZSF1 females, therefore, display various features of DKD: higher-trending blood glucose levels, hyperlipidemia, and progressive proteinuria, but not hypertension. Thus, ZSF1 female rats may be a suitable model for studying DKD without hypertension and for testing the effects of DKD-relevant drug responses in females.

## 1. Introduction

The International Diabetes Federation estimates 589 million adults currently have diabetes, and up to 40% of them develop diabetic kidney disease (DKD) [1,2]. The prevalence of DKD, especially in T2DM, has been increasing since 1990 [3,4,5]. Current treatment for DKD focuses on managing the risk factors—hyperglycemia and hypertension, and yet patients progress to end-stage renal disease (ESRD), albeit slowly, requiring dialysis or kidney transplantation. DKD is still the leading cause of ESRD. The financial burden imposed by DKD is substantial and continues to grow due to the rising prevalence of diabetes and its complications. In the US alone, diabetes and its related complications accounted for $413 billion in total medical costs and lost work in 2022, according to the CDC [6]. A recent Veterans Affairs study recounted a dramatic increase in healthcare costs alongside DKD stage progression, with the overall care costs per patient per month increasing from $1597 (stage 1) to $6999 (stage 5), and monthly costs exceeding $10,000 once the veterans started to receive long-term dialysis [7]. Moreover, numerous cases of DKD are diagnosed or categorized under other co-morbidities; consequently, most estimates grossly under-represent the actual financial burden of DKD. Thus, DKD imposes a significant health, productivity, and financial burden, which is predicted to increase in the absence of intervention [3,5]. The need for novel therapies and therapeutic targets necessitates a deeper understanding of the onset and progression of this disease.

The use of preclinical model systems is critical in understanding and characterizing DKD. Several animal models have been developed and studied, and have provided mechanistic insights into the onset and progression of DKD. However, few preclinical models accurately replicate human DKD. DKD models fall into several categories: STZ-induced models of T1DM-DKD; leptin/leptin-receptor deficiency-induced hyperphagia, obesity, and renal disease; nephrectomy-based models; and models that combine diabetes, obesity, and hypertension. The obese ZSF1 rats are an example of the last group. Developed by Genetic Models Inc. (Indianapolis, IN, USA) and first described by Tofovic et al. (2000), obese ZSF1 are the progeny of a cross between two strains heterozygous for leptin receptor mutations: lean female Zucker Diabetic Fatty rats (ZDF, +/Lepr^fa^) and male Spontaneously Hypertensive Heart Failure rats (SHHF, +/Lepr^cp^) [8]. The F1 progeny homozygous for the leptin receptor deficiency (Lepr^fa/cp^), unlike their lean heterozygous littermates, are obese, diabetic, and hypertensive, thus combining the three most common risk factors for DKD. The natural course of early renal manifestations in the obese ZSF1 rats, focusing on structural, functional, and mechanistic changes, was first described by our group [9]. Indeed, these rats develop progressively worsening renal disease that culminates in their death at the age of around a year, with signs of ESRD and heart failure. ZSF1 rats are one of the few rodent models that mimic the course of progressive human T2DM-DKD, characterized by proteinuria, GFR decline, mesangial matrix expansion, mesangiolysis, arteriolar hyalinosis, thickening of the glomerular basement membrane, and tubulointerstitial fibrosis [8,9,10,11].

It is important to note that the ZSF1 rats studied as a preclinical model, like most preclinical models, were male. The female obese ZSF1 rats were largely ignored. We found a few conference abstracts [12,13,14] and only three recent articles [15,16,17] that have investigated aspects of renal function in female obese ZSF1 rats; these studies (on up to around 24-week-old rats) found milder disease manifestations and altered renal expression of transporters. Sex-bias in the choice of preclinical models could have stemmed from several factors, including simplicity, lower costs, hormone confounding effects, historical precedence, and reports of men having an elevated risk for DKD [18,19,20,21,22,23]. Nevertheless, the bias in preclinical model choice exists and has multiple concerning consequences, including the limited generalizability of study results and the potential for overlooking sex-specific differences, which can result in the poor translation of preclinical findings to human clinical trials and contribute to health disparities [18,19,20,21,22,24]. Complicating matters further, the clinicopathological manifestations of DKD are increasingly being recognized as heterogeneous. The recognized heterogeneity in DKD manifestation warrants revisiting the existing animal models for studying DKD and testing novel models in greater detail to identify possibilities of a better fit with subgroups that share common clinical indices under the umbrella of DKD. To contribute to bridging such gaps, we aimed to study and provide a comparative overview of DKD progression in male and female obese ZSF1 rats over a longer period of time (up to around 50 weeks of age).

## 2. Materials and Methods

### 2.1. Animal Rearing and Handling

The animal handling, rearing, and procedures used in the study strictly adhered to the protocol (#22016), which was reviewed and approved by the Texas Tech University Health Sciences Center–Institutional Animal Care and Use Committee (TTUHSC-IACUC) before commencing the study. Twelve six-week-old obese ZSF1 rats (strain code: 378)—six males and six females—were acquired from Charles River Laboratories. Twelve age-matched caesarean-derived Sprague-Dawley (CD) rats (Sprague Dawley rats (CD^®^ IGS); strain code: 001; also, six males and six females) were procured from Charles River Laboratories and used as controls. The rats received different diets to accelerate the onset and progression of diabetes in the test group, with ZSF1 rats receiving a relatively higher fat diet (Purina 5008—17% calories from fat; ZDF rats were developed using the Purina 5008) and CD rats receiving control diets (Prolab RMH 2500—12% calories from fat). Food and water were available ad libitum. Rats of the same sex and strain were pair-housed and kept on a 12 h light-dark cycle. Health checks were performed daily to spot signs of pain or distress (hunching, piloerection, behavioral changes, vocalization, excess porphyrin) that might warrant a veterinary consult and, thereafter, if necessary, euthanasia. They were trained through a series of brief sessions before the low-stress procedures to minimize distress and reduce stress-related behaviors that could impact study results (adapted from [25]). Unless otherwise mentioned, all animals were included in all experiments, with a single animal as the experimental unit, resulting in a sample size of six for each group in each experiment. The sample size was chosen as the minimum size required to detect changes in the tested parameters based on a general summary from prior publications in this field and a power calculation based on being able to decipher a 20% higher systolic BP between CD (estimated to have a systolic BP of ~125 mmHg) and ZSF1 males (~150 mmHg). Rats were weighed every week. None of the animals died or required euthanasia during the study period. At the end of the study period, the animals were euthanized by CO_2_ asphyxiation followed by major organ removal (heart and kidneys) during necropsies.

### 2.2. Blood Pressure Measurements

Every rat was familiarized with the restrainers, the warming platform, and the room where blood pressure (BP) measurements were taken in three to four 15 min training sessions in the week preceding their BP recording using the Kent CODA^®^ high throughput non-invasive BP measuring system, version 4.1 (Kent Scientific Corp., Torrington, CT, USA) which uses volume pressure recording (VPR) to measure the blood pressure by determining the tail blood volume.

We adapted the protocol for BP measurements from the manufacturer’s user guide and Daugherty et al. [26]. Briefly, a BP measurement session included one to four animals, depending on the sex, strain, and size of the animals. Rats of different sexes and strains were not combined to avoid unfamiliar-smell-related changes in heart rate and BP. Once the software settings were configured, the animals were allowed to freely enter the appropriately sized holders with opaque nose cones (CODA^®^ animal holders; Kent Scientific Corp., Torrington, CT, USA). A good fit allowed the animal to be comfortable, albeit with limited movement, and its tail extended beyond the rear hatch. The animals were then placed on a warming platform (set at level 3 warming), ensuring that the entire tail rested on the warming platform. An occlusion cuff was tested for fit (slid freely but fit closely) and placed close to the base of the tail. Similarly, a VPR cuff was then placed by sliding it up the tail. The temperature of the tail was then measured using a CODA^®^ infrared thermometer (Kent Scientific Corp., Torrington, CT, USA) to ensure it fell within the acceptable range for starting the experiment (32–35 °C). The animals were monitored for signs of stress throughout the experiment, which consisted of five acclimation cycles and 20 measurement cycles, with an intercycle delay of five seconds and an occlusion pressure of 250 mmHg. The deflation time was set at 15 s, and the minimum volume at 20 μL. A successful experiment had at least 10 accepted readings; otherwise, the rat was retested the next day. Figure S1 presents an example of an acceptable reading. All BP measurement experiments were scheduled around the same time each day in a quiet room to minimize diurnal variations. Measurements were taken in a randomized order. The room temperature was controlled and essentially the same between the groups. The reported outputs include systolic and diastolic BP, mean arterial pressure (MAP), and heart rate.

### 2.3. Urinalysis

Rats were individually housed in metabolic cages for four hours with free access to food and water. The volume of the collected urine was noted, and the samples were then tested using UriScan™ 10SGL Urine Test Strips U39 (YD Diagnostics Corp., Yongin-si, Gyeonggi-do, Republic of Korea) according to the manufacturer’s instructions. All measurements were done in triplicate.

### 2.4. Blood Glucose Measurements

All rats were habituated to the blood sampling procedure from the lateral tail vein for 7–10 days to minimize animal stress and the stress-induced physiological responses that might affect blood glucose measurements. Trained rats were manually restrained. A small sterile-needle prick was made in the tail close to the lateral tail vein, just enough to draw a drop of blood, after the area was cleaned, disinfected, and dried [27,28,29]. The Bayer Ascensia Contour blood glucose monitoring system (model 9545A) was used to test blood glucose levels from a drop of blood drawn from the tail after ensuring accuracy by testing a control solution. Blood glucose was tested at 19 weeks and 48 weeks of age.

### 2.5. Statistical Analyses

The reported results represent the mean ± SD of measurements from a group. The sample size, as mentioned above, was six for each group, with exceptions indicated where applicable. Unless otherwise noted, between-group differences were examined for statistical significance using two-tailed unpaired *t*-tests. Difference between means with 95% confidence intervals (CI) and Cohen’s *d* are presented as effect size estimations. Two-tailed paired *t*-tests were used to test the effect of age for each group. Regular two-way ANOVAs were conducted to test the effects of strain, sex, and their interaction on the measurements. A *p*-value less than 0.05 denotes statistical significance. Please note that this exploratory study primarily aims to provide a comparative phenotypic characterization of male and female ZSF1 rats, thereby informing future research and generating hypotheses. The data were plotted and analyzed on spreadsheets (Microsoft Excel, version 2108) and graphed in BioRender. BioRender Graph uses R (version 4.2.2) to compute results for all statistical analyses. All figures were made in BioRender.

## 3. Results

### 3.1. Growth Parameters of ZSF1 Rats Compared with CD Rats

**Figure 1:**
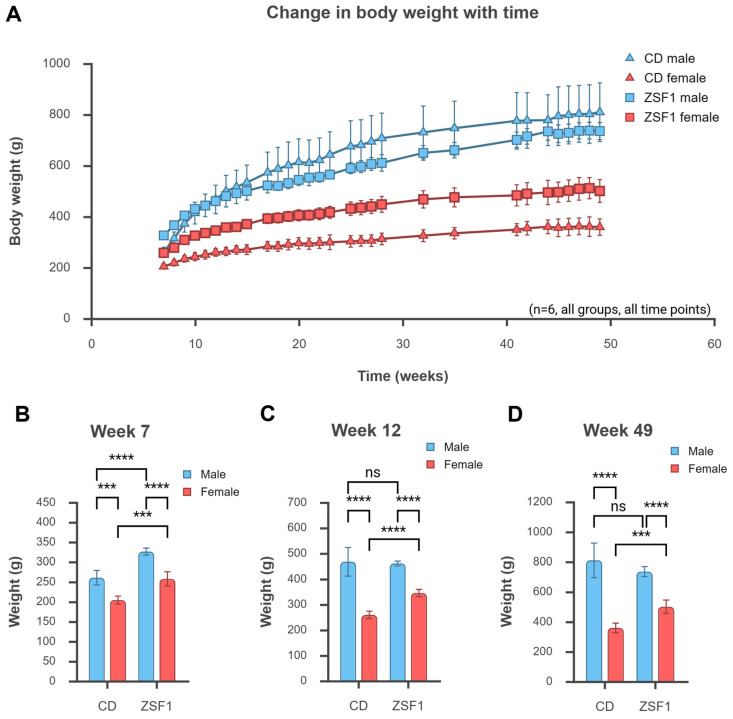
Changes in body weight with age. ZSF1 rats remain obese throughout their lives. Male CD rats became larger animals, matching the weight of ZSF1 males by 12 weeks; however, CD rats never developed obesity. Female rats were smaller and lighter than males in both the CD and ZSF1 strains. Female ZSF1 rats were always significantly heavier than female CD rats. (**A**) graphs the change in the average body weight (±SD) with age for all the test groups, while (**B**–**D**) represent between-group comparisons at 7, 12, and 49 weeks of age, respectively. *n* = 6 for every group at all time points. The results of two-tailed unpaired *t*-tests are reported in graphs (**B**–**D**) (ns: *p* > 0.05, ***: *p* ≤ 0.001, ****: *p* ≤ 0.0001). The ANOVAs reported a significant effect of strain that diminished with time (7 weeks, *p* < 0.0001; 12 weeks, *p* = 0.0043; 49 weeks, *p* = 0.2257) and a consistently significant effect of sex (7 weeks, *p* < 0.0001; 12 weeks, *p* < 0.0001; 49 weeks, *p* < 0.0001). The interaction between strain and sex became significant with time (7 weeks, *p* = 0.3211; 12 weeks, *p* = 0.0012; 49 weeks, *p* = 0.0007). Created in BioRender. Chatterjee, A. (2025) https://BioRender.com/84rdxm5 (accessed on 12 October 2025).

ZSF1 rats are known to be obese. Both male and female ZSF1 rats were heavier and fatter than the male and female CD control rats for the first 10 weeks of their lives. Female ZSF1 rats remained significantly heavier and fatter than the female CD rats throughout the experimental period (~50 weeks). However, male CD rats (especially one in the group that became the largest animal in the cohort, weighing ~1 kg by 40 weeks of age) became progressively larger, matching the weight of ZSF1 male rats by around 12 weeks of age (Figure 1A,C). Eventually, the average male CD rat weight surpassed that of ZSF1 males, but the difference was never statistically significant. The male CD rats became bigger animals; hence, the weight difference with ZSF1 males was lost, but CD rats were never obese. Figure 1A presents the change in weights of the animal groups throughout the experiment. Figure 1B–D present a graphical representation of the weights of the animals at the beginning, middle, and end of the experiment.

### 3.2. Male but Not Female ZSF1 Rats Become Hypertensive over Time

#### 3.2.1. Changes in Blood Pressure

**Figure 2:**
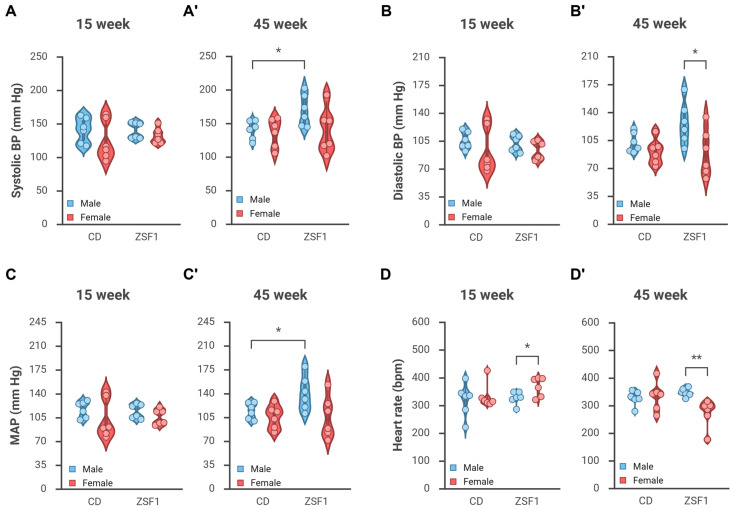
Age-related changes in blood pressure parameters. BP parameters at 15 weeks of age are comparable between the strains and the sexes (**A**–**C**). ZSF1 male rats become hypertensive with age, as indicated by the violin plots of average systolic BP (**A’**), diastolic BP (**B’**), and mean arterial pressure (**C’**). ZSF1 females also tend to have higher average blood pressure readings, and the variance within the group increases with age. (**D**,**D’**) The graphs illustrate the change in average heart rate of the test groups with age. The colored area represents the scale in the violin plots, and the box plots contained within them have whiskers extending from the 5th to the 95th percentile. *n* = 6 for every group at all time points. Only statistically significant test results from two-tailed unpaired *t*-tests are reported in the graphs (*: *p* ≤ 0.05, **: *p* ≤ 0.01). Effect size estimations from all pairwise comparisons are tabulated in Table A1. ANOVAs for 15-week parameters and 45-week systolic BP reported a non-significant effect of strain, sex, and their interaction. 45-week diastolic BP analyses reported a non-significant effect of strain (*p* = 0.2171), a significant effect of sex (*p* = 0.0137), but a non-significant interaction between strain and sex (*p* = 0.1706). 45-week MAP analyses reported a non-significant effect of strain (*p* = 0.1662), a significant effect of sex (*p* = 0.0269), but a non-significant interaction between strain and sex (*p* = 0.1744). 45-week HR analyses reported a non-significant effect of strain (*p* = 0.2357), a significant effect of sex (*p* = 0.0493), and a significant interaction between strain and sex (*p* = 0.0211). Created in BioRender. Chatterjee, A. (2025) https://BioRender.com/rwkfvy0 (accessed on 12 October 2025).

At a younger age, i.e., around 15 weeks, there was no significant difference in blood pressure between groups. BPs recorded were as follows—CD male: 140.5/105.4; CD females: 124.5/92.7; ZSF1 males: 140/101.8; ZSF1 females: 132.1/93.2 mmHg. Average systolic BP, diastolic BP, and MAPs recorded at 15 weeks of age are presented in Figure 2A–C. There was a recognizable variation between animals within the groups. Males tended to have slightly higher BP than females; however, between-group differences were not significantly different. Summary of pairwise comparisons, including effect size estimations for systolic BP, diastolic BP, and MAPs recorded at 15 weeks of age, are tabulated in Table A1. ANOVA for 15-week parameters reported a non-significant effect of strain (systolic BP *p* = 0.6627, diastolic BP *p* = 0.8274, MAP *p* = 0.9824), sex (systolic BP *p* = 0.1491, diastolic BP *p* = 0.1459, MAP *p* = 0.1371), or the interaction between strain and sex (systolic BP *p* = 0.6150, diastolic BP *p* = 0.7712, MAP *p* = 0.7023).

ZSF1 males became hypertensive with age. At around 45 weeks, ZSF1 males had elevated BP at 169.4/126.7 compared with CD males at 140/102.3, ZSF1 females at 139.7/89.3, and CD females at 136.1/90.7 mmHg (Figure 2A’–C’). Summary of pairwise comparisons, including effect size estimations for systolic BP, diastolic BP, and MAPs recorded at 45 weeks of age, are tabulated in Table A1. 45-week diastolic BP analyses revealed a significant effect of sex on BP parameters with males having higher BP than females (two-way ANOVA: systolic BP *p* = 0.1039; diastolic BP, *p* = 0.0137; MAP, *p* = 0.0270), but no significant effect of animal strain or the interaction between strain and sex.

Blood pressure tends to increase with age. As expected, BP readings at 45 weeks trended slightly higher than those at 15 weeks. All BP measurements of ZSF1 males increased significantly with age (paired one-tailed *t*-tests of 15 weeks vs. 45 weeks: systolic BP *p*-value = 0.0231; diastolic BP *p*-value = 0.0374; MAP *p*-value = 0.0310). Regardless of the statistical testing, the increases in BP measurements were also substantial enough (a 25–30 mmHg increase) to be physiologically relevant. Age-dependent changes in measured systolic BP, diastolic BP, and MAP in the animal groups are presented in Table 1.

#### 3.2.2. Change in Heart Rate

ZSF1 females exhibited age-dependent changes in heart rate. The heart rate of ZSF1 females trended higher in younger rats and lower in aging rats (Figure 2D,D’). At around 15 weeks of age, ZSF1 females had a heart rate of 367 ± 14.12 bpm, the highest amongst the groups with CD males at 319.11 ± 24.48 (unpaired *t*-test, *p* = 0.1210), CD females at 332.74 ± 18.61 (*p* = 0.1731), and ZSF1 males at 326.63 ± 9.36 (*p* = 0.0384).

In contrast, ZSF1 females had a heart rate of 273.05 ± 20.54 bpm at 45 weeks of age, indicating a substantial decrease of about 95 bpm with age (paired *t*-test, *p*-value = 0.0226) and a significantly lower heart rate than the other groups with CD males (326.50 ± 10.93; unpaired *t*-test, *p* = 0.0445), CD females (333.10 ± 21.44; *p* = 0.0707), and ZSF1 males (347.13 ± 6.21; *p* = 0.0062). Effect size estimations from between-group analyses at both time points are summarized in Table A1. No other groups showed any statistically significant changes in heart rate with age, although they seemed to trend higher with age (Table 1). Regular two-way ANOVAs were conducted for both time points. The 15-week HR analysis (similar to 15-week BP parameters) reported a non-significant effect of strain, sex, and their interaction. The 45-week HR analysis reported a non-significant effect of strain (*p* = 0.2357), a significant effect of sex (*p* = 0.0493), and a significant interaction between strain and sex (*p* = 0.0211).

### 3.3. Hyperglycemia in Male and Female ZSF1 Rats

**Figure 3:**
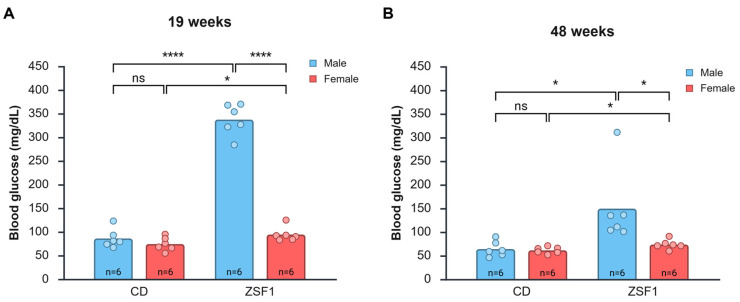
Changes in blood glucose with age. ZSF1 male rats are hyperglycemic at 19 weeks (**A**) and 48 weeks (**B**). The results of two-tailed unpaired *t*-tests are reported in graphs (ns: *p* > 0.05, *: *p* ≤ 0.05, ****: *p* ≤ 0.0001). The ANOVAs for both time points reported a significant effect of strain (19 weeks, *p* < 0.0001; 48 weeks, *p* = 0.0092), a significant effect of sex (19 weeks, *p* < 0.0001; 48 weeks, *p* = 0.0310), and a significant interaction between the two (19 weeks, *p* < 0.0001; 48 weeks, *p* = 0.0428). Created in BioRender. Chatterjee, A. (2025) https://BioRender.com/xx6cmvw (accessed on 12 October 2025).

Male ZSF1 rats exhibited significant hyperglycemia at 19 weeks of age. The average blood glucose level of 19-week-old ZSF1 male rats was 337.5 ± 33.08 mg/dL, significantly higher than that of CD males (86.17 ± 19.98; CD male vs. ZSF male: difference between means = −251.33 mg/dL (95% CI = −286.4888 to −216.1778), *p* < 0.0001, *d* = −9.1968), CD females (74.33 ± 14.49 mg/dL; *p* < 0.0001), and ZSF1 females (94.50 ± 15.50 mg/dL; *p* < 0.0001) (Figure 3A). ZSF1 females had higher blood glucose levels than CD females (CD female vs. ZSF1 female: difference between means = −20.1667 (95% CI = −39.4665 to −0.8668), *p* = 0.0422, *d* = −1.3442). Males had higher blood glucose levels than females in both strains; however, the difference in CD wasn’t statistically significant (ZSF1 male vs. ZSF1 female: difference between means = 243.0 mg/dL (95% CI = 209.7691 to 276.2309), *p* < 0.0001, Cohen’s *d* = 9.4069; CD male vs. CD female: difference between means = 11.8333 (95% CI = −10.6188 to 34.2855), *p* = 0.2675, *d* = 0.6780). However, a regular two-way ANOVA revealed a significant effect of sex (*p* < 0.0001), strain (*p* < 0.0001), and their interaction (*p* < 0.0001).

The late-stage blood glucose levels we tested were at around 48 weeks of age. The overall trends of blood glucose between the age-matched groups remained the same as in the 19-week-old groups. ZSF1 males exhibited hyperglycemia with an average blood glucose of 149.67 ± 80.47 mg/dL. Without one outlier rat (identified as a true extreme outlier by the three-times-interquartile range (3 × IQR) test), which had an exceptionally high reading of 311 mg/dL, the average ZSF1 male blood glucose level read at 117.40 ± 16.92 mg/dL. These values were still significantly higher than those of age-matched CD males (64.17 ± 16.41 mg/dL), CD females (61.50 ± 7.01 mg/dL), and ZSF1 females (73.67 ± 10.07 mg/dL). We conducted a sensitivity analysis with and without the outlier to assess how the extreme data point impacted our overall findings and determine the robustness of our conclusions. With the outlier, the comparisons yielded the following effect sizes: (a) CD male vs. ZSF male: difference between means = −85.5 mg/dL (95% CI = −160.2077 to −10.7923), *p* = 0.0289, Cohen’s *d* = −1.4723 and (b) ZSF1 male vs. ZSF1 female: difference between means = 76.0 mg/dL (95% CI = 2.2280 to 149.7720), *p* = 0.0446, Cohen’s *d* = 1.3253 (Figure 3B). Without the outlier, pair-wise comparisons yielded the following effect sizes: (a) CD male vs. ZSF male: difference between means = −53.2333 mg/dL (95% CI = −76.0270 to −30.4397), *p* = 0.0005, *d* = −3.1991 and (b) ZSF1 male vs. ZSF1 female: difference between means = 43.7333 mg/dL (95% CI = 25.1719 to 62.2948), *p* = 0.0005, *d* = 3.2274. Aged males also had higher blood glucose levels than females in both strains; however, the difference in CD still wasn’t statistically significant (CD male vs. CD female: difference between means = −2.6667 mg/dL (95% CI = −13.5663 to 18.8997), *p* = 0.722; *d* = 0.2113). ANOVA revealed a significant effect of sex (*p* = 0.0310), strain (*p* = 0.0092), and their interaction (*p* = 0.0428). Aged ZSF1 females had higher blood glucose levels than CD females (CD female vs. ZSF1 female: difference between means = −12.1667 mg/dL (95% CI = −23.3284 to −1.0049), *p* = 0.0355, *d* = −1.4022). The difference between ZSF females and CD females was comparatively smaller than the difference between the males at both time points. Nevertheless, we think the difference (12–20 mg/dL) was physiologically relevant.

Interestingly, we found a decrease in average blood glucose levels with age amongst all groups. A two-tailed paired *t*-test of all animals irrespective of group also indicated a statistically significant reduction in blood glucose levels with age (*p* = 0.0035). While the decrease in blood glucose levels among female rats didn’t reach statistical significance, the trend is quite clear. Age-dependent changes in average blood glucose levels in the animal groups are presented in Table 2. Persistent hyperglycemia, even when moderate, is pathophysiologically relevant because it promotes protein glycation, structural perturbations in immunoglobulins, and the generation of advanced glycation end-products that contribute to diabetes complications [30].

To summarize, both male and female ZSF1 rats exhibited relatively higher blood glucose levels compared with CD control rats; however, males were significantly more hyperglycemic than females. Blood glucose levels decreased with age.

### 3.4. Urinalysis of Male and Female ZSF1 Rats

**Figure 4:**
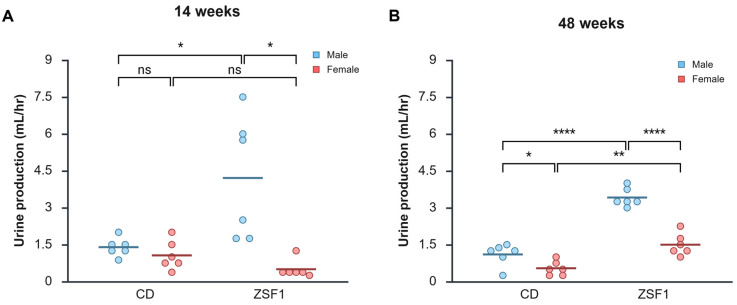
Changes in the volume of urine produced with age. ZSF1 males produce significantly more urine throughout their lives, as seen at 14 weeks (**A**) and 48 weeks (**B**). The results of two-tailed unpaired *t*-tests are reported in graphs (ns: *p* > 0.05, *: *p* ≤ 0.05, **: *p* ≤ 0.01, ****: *p* ≤ 0.0001). The ANOVAs for both time points reported a significant effect of strain (14 weeks, *p* = 0.0490; 48 weeks, *p* < 0.0001), a significant effect of sex (14 weeks, *p* = 0.0012; 48 weeks, *p* < 0.0001), and a significant interaction between the two (14 weeks, *p* = 0.0051; 48 weeks, *p* = 0.0005). Effect sizes from the pairwise comparisons are described in the results (Section 3.4). Created in BioRender. Chatterjee, A. (2025) https://BioRender.com/ts231ll (accessed on 12 October 2025).

Urinalysis of the study animals also revealed several interesting findings. Increased urine production is a well-known manifestation of diabetes. Even at the beginning of the experiment, male ZSF1 rats produced much larger volumes of urine, i.e., 4.2 ± 2.5 mL/h, compared with CD males: 1.4 ± 0.4 mL/h (CD male vs. ZSF1 male: *p* = 0.0400, difference in means = −2.8125 (95% CI = −5.4389 to −0.1861), *d* = −1.5691); CD females: 1.1 ± 0.6 mL/h (CD female vs. ZSF1 males: *p* = 0.0267; difference in means = −3.1458 (95% CI = −5.7698 to −0.5218), *d* = −1.7273); and ZSF1 females: 0.5 ± 0.4 mL/h (ZSF1 males vs. ZSF1 females: *p* = 0.0147; difference in means = 3.7083 (95% CI = 1.0819 to 6.3348), *d* = −2.0693) (Figure 4A). This trend was retained in the aging animals tested at 48 weeks. Male ZSF1 rat cohort produced the largest volume of urine (3.4 ± 0.4 mL) compared with CD males: 1.1 ± 0.5 mL/h (ZSF1 male vs. CD male: *p* < 0.0001, difference between means = 2.3125 (95% CI = 1.7788 to 2.8462), *d* = 5.5737); CD females: 0.5 ± 0.3 mL/h (ZSF1 male vs. CD female: *p* = < 0.0001, difference between means = 2.8750 (95% CI = 2.4415 to 3.3085), *d* = 8.5322); and ZSF1 females: 1.5 ± 0.4 mL/h (ZSF1 male vs. ZSF1 female: *p* < 0.0001, difference between means = 1.9167 (95% CI = 1.3850 to 2.4484), *d* = 4.6372) (Figure 4B). At 14 weeks, females of the two strains did not differ significantly in urine production (CD female vs. ZSF1 female: *p* = 0.0763, difference in means = 0.5625 (95% CI = −0.071510 to 1.1966), *d* = 1.1412) (Figure 4A). Amongst the aging female rats, ZSF1 produced significantly more urine than CD rats (ZSF1 female vs. CD female: *p* = 0.00135, difference between means = 0.9583 (95% CI = 0.4724 to 1.4443), *d* = 2.5368) (Figure 4B). Another observation supporting the excessive urination of ZSF1 males is that we had to change their cages daily due to filthy and wet bedding material, which was a departure from the usual protocol of changing every three days, which worked for all the other groups. Overall, males produced more urine than females, as expected from their larger body sizes. Paired comparisons revealed that no group, except for ZSF1 females, exhibited any significant changes in urine production over time (CD males 14 vs. 48 weeks, *p* = 0.2682; CD females 14 vs. 48 weeks, *p* = 0.1717; ZSF1 males 14 vs. 48 weeks, *p* = 0.4870). ZSF1 females, however, showed a clear trend of increasing urine production with age from 0.5 ± 0.4 mL/h to 1.5 ± 0.4 mL/h (paired *t*-test: *p* = 0.0077). Regular two-way ANOVAs conducted to examine the effects of sex and strain revealed a significant effect of sex (19 weeks, *p* = 0.0012; 48 weeks, *p* < 0.0001), a significant effect of strain (19 weeks, *p* = 0.0490; 48 weeks, *p* < 0.0001), and a significant interaction between the two (19 weeks, *p* = 0.0051; 48 weeks, *p* = 0.0005).

#### 3.4.1. Proteinuria

Both male and female ZSF1 rats exhibited slight proteinuria, even at 14 weeks of age (Table 3). Dipstick tests indicated an average of around 1.5–2 mg/mL (0.4–1 mg/mL protein levels can be expected in normal rat urine). By 32 weeks, the average proteinuria for all ZSF1 rats (males and females) amplified to ≥10 mg/mL, a level that was maintained at 48 weeks.

#### 3.4.2. Glucosuria

Male ZSF1 rats exhibited glucosuria as early as 14 weeks of age (Table 3), with an average glucose level of 500–1000 mg/dL. All female ZSF1 rats tested negative for glucosuria, except one, which had a concentration between 250 and 500 mg/dL. Notably, the wet bedding material of ZSF1 male and not female rat cages had the distinctive sweet or fruity odor recognized in uncontrolled diabetic urine. Male ZSF1 rats continued to exhibit heightened glucosuria at 32 weeks (averaging ~1500 mg/dL). However, by 48 weeks of age, glucose levels detected in their urine had reduced dramatically, with three animals showing 100 mg/dL or more glucose in urine and the remaining three animals testing negative for glucosuria. Half of the ZSF1 females tested positive for glucose in urine at 32 weeks and 48 weeks of age (~100 mg/dL). Notably, all samples of urine (from any rat at any age) tested negative for ketones.

#### 3.4.3. Indications of Infection

Elevated levels of white blood cells (WBCs) in urine, also known as pyuria, can indicate a urinary tract infection (UTI), kidney infection, or inflammation in the urinary tract. Few leukocytes were detected in the urine of 14-week-old ZSF1 male rats (Table 3). However, all 48-week-old ZSF1 male rats revealed very high numbers of leukocytes in their urine (averaging 75–500 WBC/μL of urine). All ZSF1 male urine samples tested negative for nitrites at both time points, providing strong indications of urinary tract inflammation rather than infection. Aging female ZSF1 rats also had detectable WBCs in their urine (25–75 WBCs/μL of urine at 48 weeks, but none at 14 weeks). No leukocytes or any other indications of infection were detected in the urine samples of any CD rats—male or female—at either of the tested time points. The urine collected from any rat wasn’t cloudy, but clear. It is worth noting that significant urinary protein excretion (>5 mg/mL) may lead to false-negative results in testing for leukocytes. Similarly, high glucose conc. (>2000 mg/dL) may diminish the test reaction in some leukocytes, leaving them undetected. Thus, the numbers we detected in ZSF1 rats are surely an under-representation of the actual numbers of WBCs in their urine. Nevertheless, increasing numbers of leukocytes with negative nitrite tests in urine indicate inflammation in the urinary tract in ZSF1 rats, which increases with age and is higher in males than in females.

### 3.5. Peri-Euthanasia Gross Pathology

**Figure 5:**
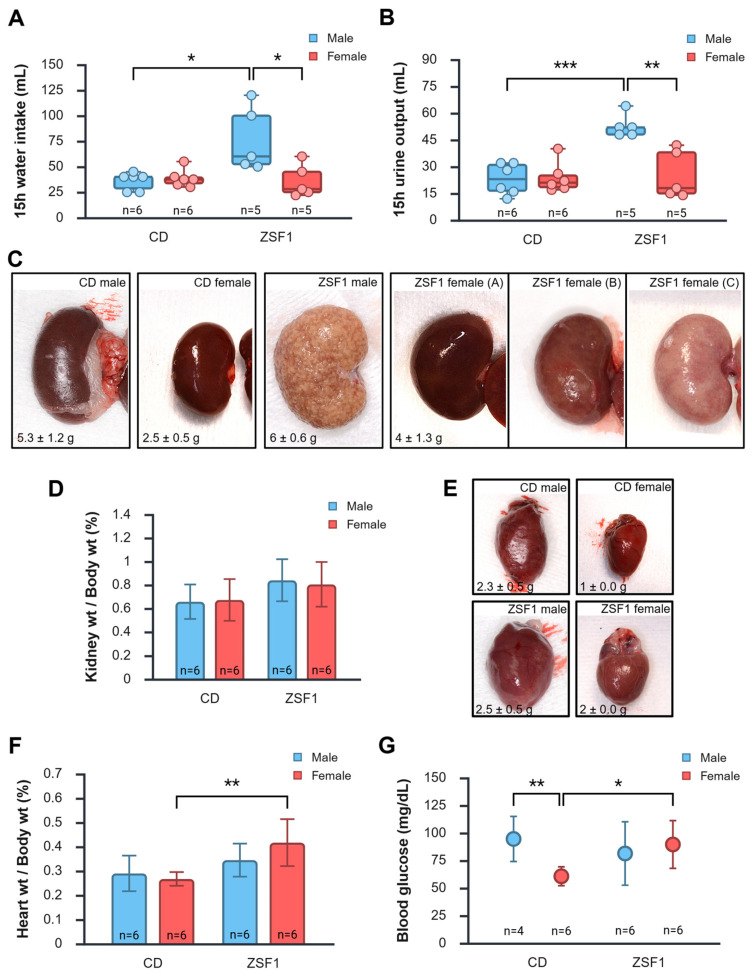
Peri-euthanasia gross cardio-renal pathology. (**A**,**B**) represent the changes in the volume of water intake (**A**) and urine produced (**B**) in 15 h between the test groups. ZSF1 males had significantly higher values for both measurements. (**C**) Representative images of kidneys indicate gross pathology in ZSF1 male and female rats. ZSF1 females had varying degrees of pathological changes. Although the ZSF1 kidneys seemed enlarged, normalized kidney weights (**D**) measured at the end of the protocol indicate no statistically significant increase in either male or female ZSF1 rats; however, the effect of strain was significant (*p* = 0.0382). (**E**) Representative images of the heart indicate gross pathology in ZSF1 male and female rats. (**F**) Normalized heart weights measured at the end of the protocol indicate a significant increase in ZSF1 females, and the effect of strain was also significant (*p* = 0.0022). (**G**) Blood glucose levels measured in heart samples were significantly lower in CD females. ANOVA indicated a non-significant effect of strain (*p* = 0.4052), a non-significant effect of sex (*p* = 0.1796), but a significant interaction between the two (*p* = 0.0347). Only the statistically significant results of two-tailed unpaired *t*-tests are reported in the graphs (**A**,**B**,**D**,**F**,**G**). *: *p* ≤ 0.05, **: *p* ≤ 0.01, ***: *p* ≤ 0.001. Created in BioRender. Chatterjee, A. (2025) https://BioRender.com/7kfm00a (accessed on 12 October 2025).

Overnight water intake and urine output were measured the day before the rats were euthanized. ZSF1 male rats drank significantly more water (76.5 ± 31.6 mL) than CD males (35.8 ± 8.6 mL), CD females (38.8 ± 8.8 mL), or ZSF1 females (36 ± 16.1 mL) (Figure 5A). The results of pairwise comparisons and estimations of effect size for end-stage water intake are as follows: (a) CD male vs. CD female: difference between means = −2.9167 (95% CI = −14.0960 to 8.2626), *p* = 0.574, *d* = −0.3356; (b) ZSF1 male vs. ZSF1 female: difference between means = 40.50 (95% CI = 3.9187 to 77.0813), *p* = 0.034, *d* = 1.6147; (c) CD male vs. ZSF1 male: difference between means = −40.6667 (95% CI = −70.8364 to −10.4970), *p* = 0.0138, *d* = −1.8464; (d) CD female vs. ZSF1 female: difference between means = 2.75 (95% CI = −14.4704 to 19.9704), *p* = 0.726, *d* = 0.2187. Similarly, the urine output for the ZSF1 male rats (52.8 ± 6.6 mL) was significantly higher than that of CD males (23.0 ± 8.7 mL), CD females (23.8 ± 8.5 mL), or ZSF1 females (25.4 ± 13.5 mL) (Figure 5B). The results of pairwise comparisons for end-stage water intake are as follows: (a) CD male vs. CD female: difference between means = −0.8333 (95% CI = −11.9505 to 10.2838), *p* = 0.871, *d* = −0.09643; (b) ZSF1 male vs. ZSF1 female: difference between means = 27.40 (95% CI = 11.9309 to 42.8691), *p* = 0.0035, *d* = 2.5833; (c) CD male vs. ZSF1 male: difference between means = −29.80 (95% CI = −40.5549 to −19.0451), *p* = 0.0001, *d* = −3.7955; (d) CD female vs. ZSF1 female: difference between means = 1.5667 (95% CI = −16.6555 to 13.5222), *p* = 0.82, *d* = −0.1422. One ZSF male and one ZSF female were found in slight distress the day before their planned euthanasia (ZSF male: hunched, porphyrin around eyes; ZSF female: lethargic, avoided touch). They were therefore excluded from the 15 h metabolic chamber housing for water intake and urine output measurements, resulting in a sample size of five for these two test groups.

Kidneys recovered from ZSF1 rats at the end of the study showed several pathological changes. The extreme obesity of ZSF1 rats was apparent during necropsy, characterized by the presence of copious amounts of visceral fat, thin and stretched muscles infiltrated with fat, and a turbid, pink appearance of the lipemic blood. CD males and females had reddish-brown, bean-shaped, firm, and smooth kidneys with no apparent indications of disease or damage. ZSF1 males had pale, enlarged, mottled, spongy, and grainy kidneys. ZSF1 females had kidneys with varying degrees of disease, faring worse than healthy CD kidneys and better than ZSF1 male kidneys: ranging from relatively smooth reddish-brown to enlarged, pale, and mottled (Figure 5C). The mean absolute kidney weight significantly varied amongst the groups, with ZSF1 males recording the heaviest kidneys (ZSF1 males: 6 ± 0.6 g) compared with CD males (5.3 ± 1.2 g; *p* = 0.2596), CD females (2.5 ± 0.5 g; *p* < 0.0001), and ZSF1 females (4 ± 1.3 g; *p* = 0.0061). Absolute kidney weight for ZSF1 females was significantly higher than that of CD females (*p* < 0.0004). But none of the between-group differences in normalized kidney weights were statistically significant (Figure 5D).

Hearts of CD rats had smooth, translucent membranes, a typical structure, and no apparent signs of disease or damage. ZSF1 rats seemed to have enlarged, paler hearts with thinner walls (Figure 5E). Absolute heart weights varied significantly amongst the groups, with ZSF1 males recording heart weights (ZSF1 males: 2.5 ± 0.5 g) comparable to CD males (2.3 ± 0.5 g; *p* = 0.5995), but heavier than CD females (1 ± 0.0 g; *p* = 0.0001), and ZSF1 females (2 ± 0.0 g; *p* = 0.0493). Absolute heart weight for ZSF1 females was significantly higher than that of CD females (*p* < 0.0001). The between-group differences in normalized heart weights remained statistically significant only for CD female vs. ZSF1 female (difference between means = −0.1495 (95% CI = −0.2413 to −0.05782), *p* = 0.0046, *d* = −2.0973) (Figure 5F). ZSF1 rats had more viscous lipemic blood. Compared with CD males (94.5 ± 20.47 mg/dL) and ZSF1 females (89.5 ± 21.65 mg/dL), CD females (60.67 ± 8.57 mg/dL) had relatively lower levels of glucose in the blood collected from the heart during necropsy (CD males vs. CD females: difference between means = 33.8333 (95% CI = 12.6230 to 55.0436), *p* = 0.0062, *d* = 2.3744; CD female vs. ZSF1 female: difference between means = −28.8333 (95% CI = −50.0137 to −7.6530), *p* = 0.0126, *d* = −1.7512) (Figure 5G). ZSF1 males (81.3 ± 28.78 mg/dL) showed no notable difference from CD males or ZSF1 females (CD male vs. ZSF1 male: difference between means = 13.1667 (95% CI = −25.5002 to 51.8336), *p* = 0.455, *d* = 0.5069; ZSF1 male vs. ZSF1 female: difference between means = −8.1667 (95% CI = −40.9257 to 24.5924), *p* = 0.591, *d* = −0.3207) (Figure 5G). Two male CD rats were used for necropsy training without having their blood glucose levels measured, resulting in a sample size of four for this group.

Overall, we found CD rats had a standard body structure, with supple joints, strong muscles, and low visceral fat. All their organs appeared normal and healthy with no signs of disease or damage. In contrast, both male and female ZSF1 rats were extremely obese with profuse visceral fat, thin muscles, enlarged fatty liver, heart, and kidneys (which were also pale and mottled; grainy in males), and lipemic blood, suggesting signs of cardio-renal and liver disease.

## 4. Discussion

In our attempt to provide a comparative overview of DKD onset and progression in male and female ZSF1 rats, we found that both male and female ZSF1 rats were overweight and obese; however, the females were more active. Male, but not female, ZSF1 rats became hypertensive with age. ZSF1 females had a higher heart rate at the beginning of their lives, which reduced significantly with age. ZSF1 males, but not females, exhibited significant hyperglycemia from an early age. However, blood glucose levels in female ZSF1 trended higher than those in CD females and males. Both ZSF1 males and females develop progressive proteinuria. ZSF1 females, therefore, displayed various etiological features of DKD: higher-trending blood glucose, hyperlipidemia, and progressive proteinuria and polyuria, but not hypertension.

Most studies compared obese ZSF1 rats with lean ZSF1 littermates to control for the genetic background. Lean ZSF1 rats have one of the leptin receptor mutations (either Lepr^fa^ or Lepr^cp^). With one normal copy of the leptin receptor, lean ZSF1 rats are less hyperphagic than obese ZSF1 rats, but they are more hyperphagic than rats with two normal copies of Lepr. Lean ZSF1 display hypertension and dysregulation in other clinicopathological parameters, albeit to a lesser degree than the obese rats [31,32]. Our choice of using CD rats as controls was intended to highlight the relatively smaller changes in the tested parameters that might remain undetectable when compared with the lean ZSF1 rats. Obese ZSF1 males and females are expected to have a similar genetic background, except for the sex chromosomes. Specifically, both sexes of the obese progeny have both copies of the leptin receptor mutated (Lepr^fa/cp^). While both sexes become hyperphagic and obese, only ZSF1 males exhibit overt hyperglycemia. ZSF1 females tend to have higher blood glucose levels than CD females; however, future studies with a larger sample size may clarify whether this difference is clinically significant. Sex plays an important role in the development and progression of hyperglycemia, with men generally being more susceptible [33,34]. Similarly, obese ZSF1 also shows a male predisposition, thus presenting an obese model to explore the sex-specific differences in nutrient absorption and metabolic differences. Babelova et al. found sex-specific differences in the expression of 103 genes in obese ZSF1 livers [35]. Female-specific genes were related to lipid metabolism and glycolysis. Male-specific genes were associated with hepatic metabolism, detoxification, and secretion, suggesting the possibility of sex-specific pharmacokinetic differences that warrant further research.

ZSF1 male rats exhibited a significant reduction in blood glucose levels with age: from ~340 mg/dL at 19 weeks to 150 mg/dL at 48 weeks to 80 mg/dL at 54 weeks, when they were euthanized. A similar reduction was seen in glucose levels in the urine. All six male ZSF1 rats exhibited glucosuria at 14 weeks, averaging 500–1000 mg/dL, which worsened at 32 weeks (averaging ~1000 mg/dL), but showed a dramatic reduction by 48 weeks (when 3/6 animals averaged ~100 mg/dL glucose in urine, and the remaining 3/6 animals tested negative). These results seem to echo the phenomenon of “burnt-out diabetes” seen in ~ 30% of patients with ESRD, who witness unexpected reductions in blood sugar levels, sometimes leading to hypoglycemia, even without active intervention or adjustment of medications [36,37]. Spontaneous resolution of hyperglycemia, normalization of HbA1c levels, and even hypoglycemic episodes are observed in these patients, alongside a further decline in kidney function [38]. Impaired renal insulin clearance and gluconeogenesis have been suggested as potential contributors [37,38], but the mechanistic details remain unexplored. To our knowledge, preclinical animal research hasn’t explored this phenomenon, possibly due to a lack of suitable model systems. We propose that male ZSF1 rats present a suitable model for studying “burnt-out diabetes”, including modified pharmacokinetics, and testing alternative drugs or refining existing treatment/management plans.

ZSF1 males, in our study, became hypertensive naturally with age, without any external intervention, e.g., a DOCA with a high salt diet or nephrectomy. This finding aligns with our previous work and other published reports [9,16,31,32]. Most studies have reported a detectable increase in systolic BP by 20 weeks of age. Notably, we did not observe any differences in BP parameters at 15 weeks of age, which is in agreement with Nguyen et al. (2023) [16]. Most published studies focus on the relatively young ZSF1 rats (8–30 weeks) and might have therefore missed the eventual late-stage changes. While not statistically significant, the trend of an increase in BP with age was evident in ZSF1 females as well. Obese ZSF1 females and males are expected to have inherited the same genetic background of mutations and alleles that predispose the parental SHHF rats to hypertension. Yet, we see a difference between BP parameters between the two sexes. Radin et al. reported that obesity secondary to leptin receptor deficiency (Lepr^cp/cp^) results in increased salt sensitivity that is mediated by endothelin in the SHHF rat [39]. On a high-salt diet, these rats showed no difference in expression of endothelial nitric oxide synthase (eNOS), neuronal NOS (nNOS), or inducible NOS (iNOS). In contrast, myocardial eNOS expression and activity increase with age and metformin treatment in SHHF rats [40,41]. While Koser et al. found no difference in phosphorylated eNOS/eNOS between lean and obese ZSF1 hearts, Franssen et al. reported a higher expression of phosphorylated eNOS monomers in obese ZSF1, similar to a trend seen in human patients with HFpEF [42,43]. Testing whether ZSF1 males and females inherit and express these changes in eNOS and endothelin, as well as similar genetic variations, remains an interesting question.

Nguyen et al. (2020) reported a reduction in heart rate over time in both anesthetized male and female obese ZSF1 rats compared to their lean littermates [44]. We observed a reduction in HR only in conscious female obese ZSF1 rats over time, measured by a non-invasive BP measurement system. Obese ZDF males exhibit a lower resting heart rate than lean ZDF males, as indicated by telemetric readings in conscious rats [45]. The methods of measurement vary considerably amongst such studies and deserve a deeper discussion to make meaningful comparisons between studies. Intra-arterial cannulation-based BP monitoring is the most physiologically accurate method of measuring BP. Implanting a radiotelemetry sensor allows for continuous and direct measurement of BP and assessment of changes in BP over time. These invasive methods of BP measurement have been considered the gold standard; however, the procedures required for these methods are costly, technically challenging, and have a non-zero failure rate, often leading to loss of animals [46,47,48]. The special housing required for continuous measurements can affect food intake, obesity, gene expression, behavior, and social acclimation, and thereby possibly BP parameters [49]. Studies exploring non-invasive methods of BP measurement have found a strong correlation between tail-cuff and intra-arterial BPs measured simultaneously in conscious mice [50,51,52] and rats [53,54,55]. For the main advantages of safety and ease, non-invasive tail-cuff-based BP measurements are recommended for comparative studies and initial experimental trials [46,47,56]. Indeed, tail-cuff plethysmography is now the most common method of non-invasive BP measurement in mice and rats. The technique relies on the tail cuff to occlude blood flow, and a choice of sensors to monitor blood pressure upon deflation of the cuff, similar to the sphygmomanometers used for BP measurements in humans. The techniques used in photoplethysmography (an optical sensor), piezo-plethysmography (a piezoelectric transducer), and volume pressure recording (VPR) vary. VPR is the simplest and most cost-effective technique, which we employed in this study. We minimized the effect of diurnal fluctuation by taking measurements at the same time of the day for all rats. We ensured the rats were trained for the procedure beforehand to minimize the effect of handling and restraints. Given that the tail-cuff method is prone to some variables and its tendency to underestimate BP (despite good correlation with telemetry) [52,53], we made every attempt to minimize environmental variations, stress to the animals, and follow the recommendations from the manufacturer and subject-matter experts [46,47,56].

ZSF1 females appear to exhibit a more delayed manifestation of the disease. Age is a significant risk factor for DKD [57,58]. A natural decline in kidney function is expected with age, but this decline is exacerbated in the presence of other risk factors like diabetes, hypertension, obesity, and heart disease. Factors such as abnormal glucose metabolism, inflammation, and oxidative stress, which drive DKD development, likely strengthen with age, leading to a faster decline in kidney function. Moreover, complex age-related factors, such as frailty, sarcopenia, and malnutrition, can influence the progression of DKD [59]. However, the pathogenesis of DKD in older individuals is poorly understood, and therefore, more research is indicated in this field.

Diabetes-induced macrovascular complications, such as coronary heart disease or stroke, are more common in women [60,61,62]. However, the information on whether sex is a risk factor for DKD is unclear [23]. Reports suggesting all three possibilities exist, i.e., a higher risk in men [63,64,65], a higher risk in women [66,67,68], or no significant difference between the sexes [69,70]. While male sex might be a risk factor for albuminuria, female sex was found to be an independent risk factor for renal insufficiency [64], highlighting the complex and heterogeneous nature of this disease. As we continue to receive more information regarding the influence of sex on the prevalence, progression, and improvement of DKD and witness changes in these influences over time, it is important to balance DKD preclinical research and include female model systems to explore sex-specific differences in the manifestation of the disease or response to treatment and improve the generalizability of study results. Both Nguyen et al. and our work find female ZSF1 rats manifest kidney disease that is milder in proportion than the same ZSF1 males [16]. We also observe a higher variability in disease manifestation in female ZSF1 compared to males. Whether this variability is a generalizable trait amongst females of other models and women remains to be seen. Obese ZSF1 males and females are expected to have inherited a similar genetic background, including all genotypic variations that influence the manifestation of metabolic syndrome. Sex-specific differences in the penetrance of mutations and genetic variations may explain the greater heterogeneity observed in female ZSF1. For example, estrogen is known to be cardioprotective; its effects are mediated by modulating NO and endothelin. A metabolite of 17β-estradiol with minimal estrogenic activity, 2-hydroxyestradiol, has been shown to attenuate obesity, metabolic syndrome, and vascular and renal dysfunction in obese male ZSF1 rats [71]. Reported effects of loss of ovarian estrogens are variable: from mild deterioration of glucose homeostasis in female ZDF rats [72] to accelerated renal disease in female obese Zucker rats [73]. Similarly, other sex hormones might also influence metabolism and renal function. Information on similar sex-specific differences relevant to DKD onset and progression remains unknown and, hopefully, a target for future research, where ZSF1 rats will play an important role.

Limitations of our study include the lack of renal histology, blood and urine chemistries, and the direct measurement of renal functional impairment (GFR/UACR). Nevertheless, we believe our work provides valuable insights that demonstrate the disease manifestation, particularly in the previously less characterized aging and female ZSF1 rats.

## 5. Conclusions

While the clinical features of most disease states and their treatment responses are assumed to be similar in both sexes, anecdotal evidence suggested otherwise, which incentivized the scientific community, in general, and the National Institutes of Health, in particular, to require the study of both sexes in clinical and experimental research. In human DKD, scientific documentation of the sex differences in the expression of clinical parameters is very limited, notwithstanding the paucity of literature in animal models of DKD. In this study, we describe the sex differences in the phenotypic expression of diabetes, hypertension, and urinary parameters in male and female ZSF1 rats (a model of T2DM with nephropathy) compared to CD rats. Our observations suggest significant differences in obesity, hypertension, and hyperglycemia, as these were more dominant in male ZSF1 rats compared to females. It is noteworthy that despite a similar genetic background and environmental conditions, the expression of the physical characteristics in male and female rats differed significantly. While several explanations could be invoked to explain such differences, including the influence of sex hormones and differential penetration of genetic mutations in males and females, these findings warrant larger and more detailed studies to understand the sex differences and the pathophysiological basis for such observations.

## Figures and Tables

**Table 1:** Blood pressure parameters of ZSF1 males and females with age.

	Groups	15 Weeks	45 Weeks	*p*-Value *
Systolic BP (mmHg)	CD male	140.51 ± 7.72	139.98 ± 5.67	0.9524
	CD female	124.50 ± 12.17	136.13 ± 8.73	0.4030
	ZSF1 male	139.96 ± 4.91	169.39 ± 9.69	**0.0463**
	ZSF1 female	132.10 ± 4.74	139.69 ± 13.60	0.6704
Diastolic BP (mmHg)	CD male	105.42 ± 4.43	102.28 ± 5.11	0.2887
	CD female	92.68 ± 11.82	90.68 ± 6.37	0.8846
	ZSF1 male	101.78 ± 4.07	126.67 ± 10.76	0.0749
	ZSF1 female	93.20 ± 4.76	89.35 ± 12.04	0.8070
MAP (mmHg)	CD male	116.78 ± 4.91	114.49 ± 4.82	0.6137
	CD female	102.90 ± 11.90	105.52 ± 6.97	0.8456
	ZSF1 male	114.16 ± 4.30	140.54 ± 10.36	0.0620
	ZSF1 female	105.84 ± 4.61	105.79 ± 12.50	0.9973
Heart rate (bpm)	CD male	319.11 ± 24.48	326.50 ± 10.93	0.6728
	CD female	332.74 ± 18.61	333.10 ± 21.44	0.9898
	ZSF1 male	326.63 ± 9.36	347.13 ± 6.21	0.1550
	ZSF1 female	367.00 ± 14.12	273.05 ± 20.54	**0.0226**

* *p*-values are the result of paired two-tailed *t*-tests for each group, testing the null hypothesis that BP measurements remain unchanged over time. *p*-values set in boldface indicate statistical significance. The sample size was six in each group, and the reading for each animal was an average of at least ten successful measurements (for more details, see Section 2.2). Data is presented as mean ± SEM.

**Table 2:** Blood glucose levels of ZSF1 males and females with age.

	Groups	19 Weeks	48 Weeks	*p*-Value *
Average blood glucose levels (mg/dL)	CD male	86.17 ± 19.98	64.17 ± 16.41	**0.0356**
	CD female	74.33 ± 14.49	61.50 ± 7.01	0.1251
	ZSF1 male	337.5 ± 33.08	149.67 ± 80.47	**0.0083**
	ZSF1 female	94.50 ± 15.50	73.67 ± 10.07	0.0508

* *p*-values are the result of paired two-tailed *t*-tests for each group, testing the null hypothesis that blood glucose measurements remain unchanged over time. *p*-values set in boldface indicate statistical significance.

**Table 3:** Results of urinalyses.

	Groups	14 Weeks	32 Weeks	48 Weeks
Proteinuria	CD male	±	±	±
	CD female	±	±	±
	ZSF1 male	++	++++	++++
	ZSF1 female	++	++++	+++
Glucosuria	CD male	−	−	−
	CD female	−	−	−
	ZSF1 male	+++	++++	+
	ZSF1 female	+	+	+
Leukocytes	CD male	−	NT	−
	CD female	−	NT	−
	ZSF1 male	+	NT	+++
	ZSF1 female	−	NT	+

Abbreviations and symbols: −, negative. ±, trace amounts of protein detected, which fall within expected levels in rat urine (≤1 mg/mL). +, ≤ 100 mg/dL glucose or 25–75 leukocytes/μL. ++, 1–3 mg/mL protein or 100–500 mg/dL glucose. +++, 3–10 mg/mL protein or 500–1000 mg/dL glucose OR 75–500 leukocytes/μL. ++++, >10 mg/mL protein or >1000 mg/dL glucose. NT, not tested.

## Data Availability

The original contributions presented in this study are included in the article/Supplementary Material. Further inquiries can be directed to the corresponding author.

## Supplementary Materials

The following supporting information can be downloaded at: https://www.mdpi.com/article/10.3390/life15101627/s1, Figure S1: An example of an acceptable reading from the BP measurements.

## Abbreviations

The following abbreviations are used in this manuscript:

ANOVA	Analysis of variance
BP	Blood Pressure
CD	caesarean-derived
CDC	Centers for disease control and prevention
CI	Confidence interval
DKD	Diabetic kidney disease
DOCA	Deoxycorticosterone acetate
eNOS	Endothelial NOS
ESRD	End-stage renal disease
GFR	Glomerular filtration rate
HFpEF	Heart failure with preserved ejection fraction
HR	Heart rate
IGS	International genetic standardization program
iNOS	Inducible NOS
IQR	Interquartile range
MAP	Mean arterial pressure
nNOS	Neuronal NOS
NOS	Nitric oxide synthase
NT	Not tested
RMH	Rats, mice, and hamsters
SD	Standard deviation
SEM	Standard error of mean
SHHF	Spontaneously hypertensive heart failure
T2DM	Type 2 diabetes mellitus
UACR	Urine albumin-creatinine ratio
UTI	Urinary tract infection
VPR	Volume pressure recording
WBC	White blood cells
ZDF	Zucker diabetic fatty
ZSF1	Zucker fatty/Spontaneously hypertensive heart failure F1 hybrid

## Appendix A

**Table A1:** Summary of pairwise comparisons of BP parameters and HR measured at 15 and 45 weeks of age (unpaired *t*-tests; *p*-values set in boldface indicate statistical significance).

Measurement	Comparison	Difference Between Means	95% Confidence Interval	*p*-Value	Cohen’s *d*
Systolic BP (mmHg) at 15 weeks	CD male vs. CD female	16.0077	−16.1007 to 48.1161	0.293	0.6413
	ZSF1 male vs. ZSF1 female	7.8625	−7.3355 to 23.0605	0.276	0.6655
	CD male vs. ZSF1 male	0.5435	−19.8451 to 20.9321	0.954	0.03429
	CD female vs. ZSF1 female	−7.6017	−36.6918 to 21.4884	0.573	−0.3362
Systolic BP (mmHg) at 45 weeks	CD male vs. CD female	3.8435	−19.3548 to 27.0418	0.72	0.2131
	ZSF1 male vs. ZSF1 female	29.6964	−7.5212 to 66.9140	0.106	1.0264
	CD male vs. ZSF1 male	−29.4159	−54.4443 to −4.3875	**0.0257**	−1.5119
	CD female vs. ZSF1 female	−3.5630	−39.5754 to 32.4494	0.83	−0.1273
Diastolic BP (mmHG) at 15 weeks	CD male vs. CD female	12.7373	−15.3886 to 40.8632	0.337	0.5826
	ZSF1 male vs. ZSF1 female	8.5833	−5.3765 to 22.5432	0.201	0.79010
	CD male vs. ZSF1 male	3.6336	−9.7688 to 17.0361	0.559	0.3488
	CD female vs. ZSF1 female	−0.5203	−28.9161 to 27.8755	0.968	−0.02357
Diastolic BP (mmHG) at 45 weeks	CD male vs. CD female	11.5969	−6.6040 to 29.7978	0.186	0.8197
	ZSF1 male vs. ZSF1 female	37.3191	1.3423 to 73.2960	**0.0434**	1.3344
	CD male vs. ZSF1 male	−24.3935	−50.9389 to 2.1520	0.0678	−1.1821
	CD female vs. ZSF1 female	1.3288	−29.0183 to 31.6758	0.924	0.05633
MAP (mmHg) at 15 weeks	CD male vs. CD female	13.8777	−14.8004 to 42.5558	0.306	0.6225
	ZSF1 male vs. ZSF1 female	8.3213	−5.7224 to 22.3650	0.216	0.7622
	CD male vs. ZSF1 male	2.6184	−11.9249 to 17.1617	0.697	0.2316
	CD female vs. ZSF1 female	−2.9381	−31.3661 to 25.4900	0.823	−0.13210
MAP (mmHg) at 45 weeks	CD male vs. CD female	8.9702	−9.9066 to 27.8470	0.315	0.6113
	ZSF1 male vs. ZSF1 female	34.7522	−1.4113 to 70.9157	0.0579	1.2362
	CD male vs. ZSF1 male	−26.0458	−51.4944 to −0.5972	**0.0458**	−1.3166
	CD female vs. ZSF1 female	−0.2638	−32.1464 to 31.6188	0.986	−0.01064
Heart rate (bpm) at 15 weeks	CD male vs. CD female	−13.6318	−82.1453 to 54.8817	0.667	−0.25510
	ZSF1 male vs. ZSF1 female	−40.3701	−78.1196 to −2.6205	**0.0384**	−1.3757
	CD male vs. ZSF1 male	−7.5227	−65.9253 to 50.8800	0.78	−0.1657
	CD female vs. ZSF1 female	−34.2609	−86.3019 to 17.7801	0.173	0.8469
Heart rate (bpm) at 45 weeks	CD male vs. CD female	−6.6036	−60.2145 to 47.0073	0.789	−0.1585
	ZSF1 male vs. ZSF1 female	74.0771	26.2554 to 121.8988	**0.00621**	1.9927
	CD male vs. ZSF1 male	−20.6311	−48.6419 to 7.3797	0.132	−0.9475
	CD female vs. ZSF1 female	60.0496	−6.1050 to 126.2042	0.0707	1.1677
