# Supplementary figures and images for: An Overview of Sex-Based Differences in the Onset and Progression of DKD in the Well-Known Model, ZSF1 Rats
Source: Life (Basel). 2025 Oct 18;15(10):1627. doi: 10.3390/life15101627 (PMC12565004; doi:10.3390/life15101627)

## Supplemental Figure

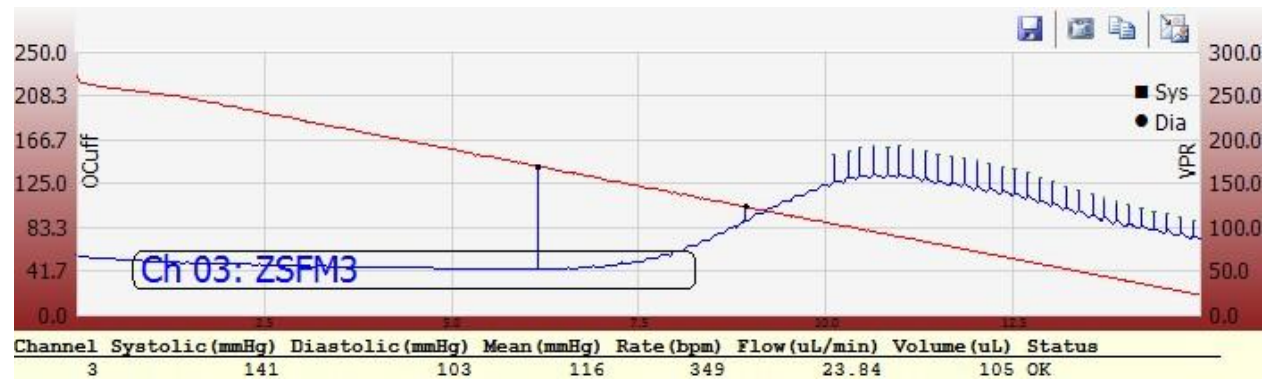

Figure S1. An example of an acceptable reading from the BP measurements

Supplement: Supplementary file 1 [file life-15-01627-s001.zip › life-3899952-supplementary.pdf]
